# Clinical application of iodine-eluting stent in patients with advanced esophageal cancer

**DOI:** 10.3892/ol.2013.1466

**Published:** 2013-07-15

**Authors:** ZHENBO DAI, DEJUN ZHOU, JIANZHANG HU, LEI ZHANG, YUNSHOU LIN, JING ZHANG, FENGLING LI, PENG LIU, HUA LI, FULIANG CAO

**Affiliations:** 1 Key Laboratory of Cancer Prevention and Therapy, Endoscopy Center, Tianjin Medical University, Cancer Institute and Hospital, Tianjin 300060, P.R. China; 2 Tianjin Lung Cancer Center, Department of Thoracic Surgery, Tianjin Medical University, Cancer Institute and Hospital, Tianjin 300060, P.R. China; 3 Tianjin Medical University, Tianjin 300070, P.R. China

**Keywords:** esophageal cancer, stent

## Abstract

The aim of the present study was to compare the clinical effectiveness of an iodine-eluting stent with a conventional stent in patients with advanced esophageal cancer. Patients with malignant esophageal cancer were randomly assigned to receive a conventional stent (group A) or an iodine-eluting stent (group B). Following implantation, the relief from dysphagia, survival time, routine blood tests, thyroid function examination and complications were compared in the two groups. Groups A and B consisted of 36 and 31 patients, respectively. The mean value that the dysphagia score decreased by was significantly lower in group A (0.83) compared with group B (1.65). The median survival time was longer in group B compared with group A (P=0.0022). No significant differences were observed in the severe complications between the two groups (P=0.084). The iodine-eluting esophageal stent is a relatively safe, feasible and effective treatment for malignant esophageal strictures.

## Introduction

Esophageal cancer may result in stenosis and obstruction or a fistula combined with stenosis (1). Older patients who decline to have surgery and patients with post-operative stenosis comprise ~50% of all patients with advanced esophageal cancer (2–4). Dysphagia is the predominant symptom exhibited by patients with inoperable esophageal cancer. To relieve the dysphagia and improve the quality of life of patients with esophageal cancer, stent placement is a widely-accepted option for palliation of the symptoms that are caused by esophageal strictures (5–8).

The conventional metal stent only provides palliative treatment through mechanical support to improve the eating ability of a patient. In recent years, an esophageal stent loaded with iodine 125 (^125^I) seeds has been developed (9,10). This form of irradiation stent inhibits tumor growth by administering continuous low-dosage irradiation from the ^125^ seeds. However, the ^125^I seeds must be installed in the esophageal stent every time. To make the treatment easier to use, the iodine-eluting esophageal stent was developed. This is a new type of esophageal nitinol stent with a polyurethane membrane uniformly covered with ^125^I. In the present study, the conventional stent alone was compared with the iodine-eluting stent in treating malignant esophageal strictures in esophageal cancer.

## Materials and methods

### Patients

A total of 71 consecutive patients with malignant esophageal strictures were enrolled for selective intraluminal stent placement between April 2008 and December 2010. However, four patients were lost to follow-up. The specific inclusion criteria for stent placement was as follows: i) A histopathological diagnosis using an endoscopic biopsy confirming that the tumor was esophageal cancer; ii) tumor invasion or compression resulting in esophageal luminal stenosis or occlusion; iii) an expected survival time of more than one month; iv) physical fitness (Karnofsky) score ≥50; v) no serious heart, lung, hematological, nervous system, liver or kidney dysfunction; and vi) no acute infection. No patients received chemotherapy or radiotherapy treatment prior to, concurrently with or following stent placement. Approval for the study was obtained from the ethics committee of Tianjin Cancer Institute and Hospital, and informed consent was obtained from all patients. The exclusion criteria included acute infection, severe cardiovascular or mental illness and evidence of multiple small-bowel obstructions. Four patients were lost to follow-up. The remaining patients were randomly assigned into two groups, those who received a conventional stent (group A; n=36) and those who received an iodine-eluting esophageal stent (group B; n=31). No significant differences were observed in gender, age, vital signs or pain and dysphagia grades between the two groups prior to the stent placement (Table I).

### Stent

**Figure 1:**
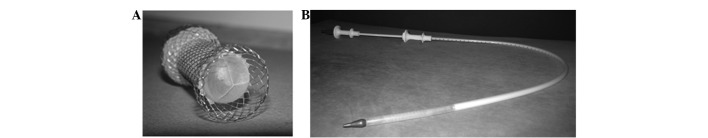
(A) Radioactive stent and (B) its auxiliary equipment.

The iodine-eluting esophageal stent was composed of two parts: An esophageal nitinol stent and a polyurethane membrane that was uniformly covered with ^125^I. All esophageal stents were produced by Anhui Jinmin Medical Instruments Co., Ltd. (Tianchang, Anhui, China; Fig. 1). The half-life of the ^125^I seeds was 59.6 days. The radiation dose was determined on the basis of the size of the individual tumor, according to clinical studies, and the activity for clinical use was 5–13.5 mCi (9–10).

### Stent placement

Prior to stent placement, the site, degree and length of the obstruction were assessed using a conventional upper gastrointestinal (GI) endoscope, computerized tomography (CT) and/or a water-soluble contrast fluoroscopic study. The type, size and length of the stent were chosen according to the measured length of the obstruction. The length of the stent was chosen to be at least an additional 2 cm on either side of the proximal and distal extent of the strictures or fistula. The two types of stents were placed in the same way and the esophagus was able to selectively expand according to the extent of the esophageal strictures.

It should be noted that nickel-titanium shape-memory alloy stents will soften when encountering cold temperatures. The temperature of the stent should be >37ºC for one week in order to allow it to form a shape. After one week, the stent does not change in response to cold temperatures. To avoid the shifting or dropping of the stent, cold or rough foods were prohibited for one week following the stent placement.

### Observation

The patients from groups A and B underwent an esophagography 1–3 days after the stent placement in order to verify the position and patency of the stent. The patients were instructed not to eat solid food until the stent had fully expanded. Following the procedure, routine blood tests, thyroid function examinations, barium meal tests, endoscopies, chest CT scans and plain X-rays were ordered at regular intervals to check for complications.

The scores of the dysphagia and pain grades and the condition of the granulation tissue were recorded for all patients pre- and post-stent placement. To assess the clinical improvement, the dysphagia score prior to and following the procedure was graded on a scale of 0–4, according to the CIRSE guidelines (11) as follows: Grade 0, normal diet; grade 1, ability to swallow solid food; grade 2, ability to swallow semi-solids only; grade 3, ability to swallow liquids only; and grade 4, complete dysphagia. The pain score was graded on a scale of 0-III as follows: 0, feeling no pain; I, mild pain and uninterrupted sleep; II, moderate pain that is not tolerable and requires pain medication and sleep disturbance; and III, severe pain and severe sleep disturbance that requires the treatment to be stopped. The condition of the granulation tissue was graded as no proliferation, mild proliferation or significant proliferation.

### Statistical analysis

The statistical analyses were performed using SAS software, version 9.0 (SAS Institute, Cary, NC, USA). The numeric data of the ages of the patients were examined using the Student’s t test, whereas other characteristics of the patients prior to the stent placement were analyzed using the χ^2^ test. The comparison of the side-effects and complications associated with the stent placement between the two groups was also analyzed using the χ^2^ test. The log-rank test was used for the evaluation of the patient survival time. P<0.05 was considered to indicate a statistically significant difference.

## Results

### Relief of esophageal obstruction

**Figure 2:**
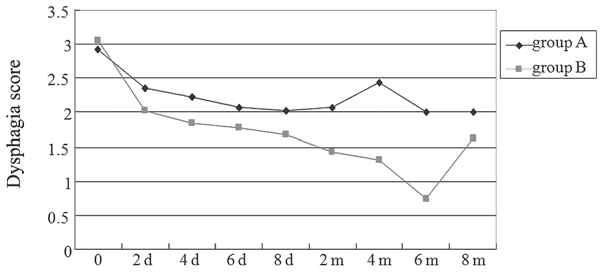
Mean dysphagia score during follow-up. The dysphagia grades significantly improved in the two groups and the mean decreased value of dysphagia score was 0.83 in group A and 1.65 in group B. There was a significant difference between the two groups (P=0.002; Student’s t-test). d, days; m, months.

The dysphagia scores improved in groups A and B, and eight days after the stent placement, there were no significant differences between the two groups (P=0.212, Student’s t-test; Fig. 2) At two months post-procedure, the mean value that the dysphagia score had decreased by was 0.83 in group A and 1.65 in group B, and a significant difference was observed between the two groups (P=0.002; Student’s t-test). At two months after the stent placement, 16 patients in group A and 11 in group B were evaluated using a gastroendoscopy examination. Hyperplasia of the granulation tissue was noted at each end of the stent, but particularly the proximal end, in all 27 patients. Hyperplasia of the granulation tissue was more evident in the patients in group A than in those of group B (P=0.007; Cochran-Mantel-Haenszel test).

### Survival

**Figure 3:**
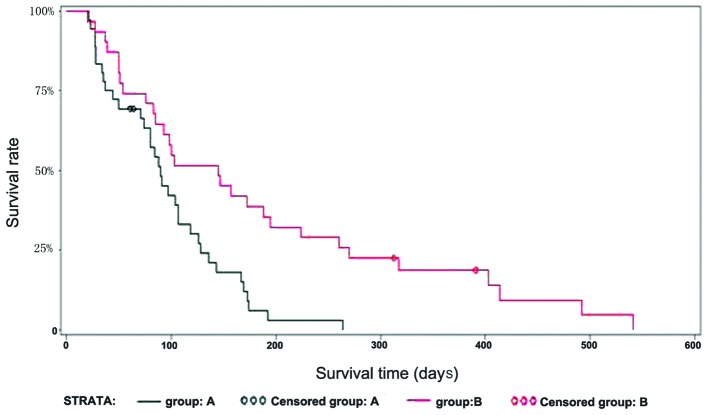
Kaplan-Meier curves. Comparison of overall survival between group A (a conventional covered stent) and group B (an iodine-eluting esophageal stent). The median overall survival period was longer in group B compared with that in group A; 145 (95% CI, 85–195) days vs. 90 (95% CI, 74–107) days, respectively.

The median survival time was longer for group B patients than group A patients [145 days (95% CI, 85–195) vs. 90 days (95% CI, 74–107), respectively], which demonstrated a significant difference between the two groups (P=0.0022, log-rank test; Fig. 3).

### Side-effects, complications and security assessment

No severe procedure-related complications occurred in any of the cases. Severe complications occurred in 11 patients in group A (28.9%) and 4 in group B (12.1%), and no significant differences were identified between the two groups (P=0.144; Fisher’s exact test). The majority of patients felt pain in the rear of the sternum following the stent placement and 25 patients (16 patients in group A and nine patients in group B) complained of severe chest pain, which was palliated using narcotic analgesics. Only one patient felt severe pain and ceased stent treatment. The degree of chest pain between the two groups was not significantly different. Tracheoesophageal fistulae occurred in three patients (two patients in group A and one in group B). Hemorrhages occurred in seven patients (five patients in group A and two in group B), but no patients succumbed due to an acute massive hemorrhage.

Prior to the stent placement, routine blood tests and thyroid function examinations were performed on the patients in groups A and B and there were no significant differences at this point or at two months post-procedure between the two groups (Table II).

## Discussion

Since malignant esophageal cancer has no specific symptoms in its early stage, 60–80% of esophageal cancers are diagnosed at the middle or advanced stage of the disease (12). Surgery is not a viable option for these patients, but a metal stent may be used as a treatment option to relieve the dysphagia, thus improving the quality of life of the patient. Compared with other treatments for esophageal strictures, the metal stent placement procedure has shown favorable characteristics. The procedure is relatively simple, rapidly effective and generally well-tolerated (13,14).

Conventional stent placement alone does not offer any therapeutic effects on the esophageal cancer itself and is used only for mechanical support and obstruction relief. Certain studies have shown that a self-expandable stent loaded with ^125^I seeds is a safe and effective treatment for esophageal cancer (9,10). However, the ^125^I seeds must be installed in the esophageal stent every time the procedure is performed. Furthermore, although the ^125^I seeds are uniformly placed in the stent, the irradiation caused by the seeds is not distributed evenly. To overcome these shortcomings, the iodine-eluting esophageal stent was developed.

In the present study, the dysphagia score was improved greatly in groups A and B, and eight days following the stent placement, there were no significant differences between the two groups. At two months post-procedure, the mean value that the dysphagia score decreased by was 0.83 in group A and 1.65 in group B, and a significant difference was observed between the two groups. The dysphagia score improved significantly in group B. The reason for restenosis may be due to the tumor tissue and/or hyperplasia of the granulation tissue growing into the stent from the superior margin at the two ends of the stent. A total of 16 patients in group A and 11 in group B underwent a gastroendoscopy examination at two months post-procedure. Restenosis due to the regrowth of the tumor tissue into the stent did not occur in any of the patients. However, hyperplasia of the granulation tissue was noted at each end of the stent, particularly at the proximal end, in all 27 patients. Hyperplasia of the granulation tissue was more evident in patients of group A than those of group B. The endoscopic examinations demonstrated that the iodine-eluting esophageal stent had partial inhibitory effects on the tumor and the hyperplasia of the granulation tissue. Furthermore, there was a significant improvement in the survival of the patients, with a median survival time of 145 days in group B vs. 90 days in group A. This difference was statistically significant, indicating the therapeutic advantages of the iodine-eluting esophageal stent.

The possible complications following the implantation of the esophageal stent include hemorrhage, perforation and tracheoesophageal fistulae (15–17). Severe complications occurred in 11 patients in group A (35.5%) and four in group B (11.1%), and there were no differences between the two groups. Hemorrhaging has been reported in 3–8% of all stent patients and is usually self-limited (1). Guo *et al*(9) reported that hemorrhaging occurred in 16 patients (30%) in two groups studied during implantation and follow-up. In the present study, hemorrhaging occurred in seven patients (five patients in group A and two in group B), but no patients succumbed due to an acute massive hemorrhage. Esophageal perforations or tracheoesophageal fistulae have been shown to occur in 2.7–7.3% of patients following stent placement (18–20). Although the occurrence of such a complication may be increased with an iodine-eluting stent due to the radiation effect on the esophageal wall, tracheoesophageal fistulae occurred in three patients in the present study (two patients in group A and one in group B) with no significant differences between the two groups, indicating that an esophageal perforation or a tracheoesophageal fistula is mainly caused by the shearing action of the edge of the esophageal stent and necrosis of the tumor.

The radiation effect of the iodine-eluting esophageal stent may have an effect on thyroid function and blood characteristics. In the present study, no significant differences were observed between the two groups in the evaluation of the routine blood tests and thyroid function examinations prior to the stent placement or at two months later. This indicated that the radioactivity of the iodine-eluting esophageal stent has little effect on thyroid function and routine blood results.

There are certain limitations to the present study. First, the irradiation dose of the iodine-eluting esophageal stent was only selected using the length of the tumor due to the lack of sophisticated measuring techniques for esophageal cancer. Second, the quality of life, which is a significant measure of outcome for the palliative treatment of malignancies, including inoperable esophageal cancer, was not measured in the present study.

In conclusion, the present data show the iodine-eluting esophageal stent to be a relatively safe, feasible and effective treatment for esophageal stenosis caused by advanced esophageal carcinoma. Treatment is believed to be improving continuously with the development of advanced materials and techniques, and the long-term prognosis and effectiveness of the iodine-eluting stent requires further evaluation through further observation and research.

## Figures and Tables

**Table I:** Background characteristics of patients prior to stent placement.

Characteristic	Group A	Group B	P-value
Age (years)^a^	71.26±8.93	68.13±10.44	0.191^b^
Gender			0.529^c^
Male	28	26	
Female	8	5	
Heart rate (bpm)^a^	77.40±7.04	75.03±9.17	0.241^b^
Respiratory rate (bpm)^a^	18.61±1.63	18.06±1.77	0.192^b^
SBP (mmHg)^a^	118.67±17.83	117.74±12.19	0.803^b^
DBP (mmHg)^a^	74.56±8.72	76.35±9.34	0.418^b^
Temperature (ºC)^a^	36.49±0.43	36.60±0.26	0.235^b^
Dysphagia grade			0.327^d^
1	2	0	
2	4	3	
3	25	23	
4	5	5	
Pain grade, n (%)			0.070^d^
0	11 (52.4)	17 (77.3)	
I	9 (42.9)	5 (22.7)	
II	1 (4.8)	0 (0.0)	
III	0 (0.0)	0 (0.0)	

a Data are presented as the mean ± standard deviation.

The ^b^Student’s t-test, ^c^χ^2^, and ^d^Cochran-Mental-Haenszel tests were used.

SBP, systolic blood pressure; DBP, diastolic blood pressure.

**Table II:** Blood regular and thyroid function examination at two months post-stent insertion

Examination	Group A	Group B	P-value
Hg (g/l)^a^	117.82±14.53	127.44±31.50	0.398^b^
WBC (x10^9^/l)^a^	7.81±2.00	7.90±2.89	0.924^b^
Plt (x10^9^/l)^a^	278.59±84.56	295.00±127.57	0.697^b^
TT3			0.580^c^
Normal	5	5	
Abnormal, no clinical significance	4	1	
Abnormal, clinical significance	0	0	
TT4			1.000^c^
Normal	9	6	
Abnormal, no clinical significance	0	0	
Abnormal, clinical significance	0	0	
TSH			0.967^d^
Normal	14	7	
Abnormal, no clinical significance	0	1	
Abnormal, clinical significance	1	0	
FT4			1.000^c^
Normal	10	7	
Abnormal, no clinical significance	1	0	
Abnormal, clinical significance	0	0	
FT3			1.000^c^
Normal	7	4	
Abnormal, no clinical significance	4	2	
Abnormal, clinical significance	0	0	

a Data are presented as the mean ± standard deviation.

The ^b^Student’s t-test, ^c^Fisher’s exact test and ^d^Cochran-Mental-Haenszel test were used.

Hg, hemaglobin; WBC, white blood cells; Plt, platelets; TT3, total triiodothyronine; TT4, total thyroxine; FT4, free thyroxine; FT3, free triiodothyronine; TSH, thyroid-stimulating hormone.
